# Do We Need Preoperative Antibiotics in Common General Pediatric Surgery Procedures?

**DOI:** 10.7759/cureus.65805

**Published:** 2024-07-30

**Authors:** Takayuki Fujii, Aya Tanaka, Hiroto Katami, Ryuichi Shimono

**Affiliations:** 1 Pediatric Surgery, Kagawa University, Takamatsu, JPN

**Keywords:** surgical site infection, umbilical hernia, inguinal hernia, undescended testis, preoperative antibiotics

## Abstract

Background

There are limited studies on the necessity of preoperative antibiotics in surgeries for undescended testis (UDT), inguinal hernia (IH), and umbilical hernia (UH) in children. Here, we investigated the relationship between preoperative antibiotic use and surgical site infection (SSI) incidence in surgeries for UDT, IH, and UH in children.

Methods

Patients who underwent surgery for IH were subdivided based on the surgical form into those who underwent (i) open IH (OIH) repair and (ii) laparoscopic percutaneous extraperitoneal closure (LPEC). Medical records of patients who underwent surgeries for UDT and IH or UH were retrospectively examined. The SSI incidence was compared between patients receiving and not receiving preoperative antibiotics. In patients who underwent surgery for UH or LPEC, the relative risk of SSI postoperatively in the inguinal region (including surgery for UDT and OIH repair) was examined.

Results

In total, 926 patients with 1389 wounds were included in this study. SSI rates in patients who underwent surgeries for UDT and UH, OIH repair, and LPEC were 0.2% and 2.7%, 0.3%, and 0.4%, respectively. These rates were not significantly different between patients receiving and not receiving preoperative antibiotics. In patients who underwent surgery for UH, the relative risk of SSI was statistically significant at 9.8 compared with that in patients who underwent surgeries in the inguinal region (95% CI = 1.3-74; p = 0.013).

Conclusions

Preoperative antibiotics are unnecessary in surgeries for UDT and OIH repair. Patients undergoing surgery for UH should be given extensive care as they are at a high risk of SSI.

## Introduction

The surgical site infection (SSI) incidence is relatively high and may result in longer hospital stays, increased hospital charges, and complications [1]. Therefore, appropriate prophylaxis for SSI is a must. The Centers for Disease Control and Prevention (CDC)’s 1999 guideline for preoperative antibiotics was revised in 2017, but it briefly mentioned pediatric patients [2,3]. Although other guidelines for surgeries for undescended testis (UDT) and inguinal hernia (IH) have been published by the American Urological Association and the European Association of Urology, both sets of guidelines focus on adults and exclude pediatric diseases [4,5]. In 2015, the Japanese Urological Association published guidelines, which recommended the administration of the first- and second-generation cephalosporin antimicrobial agent in surgery performed while maintaining a closed urinary tract in children [6]. Conversely, preoperative antibiotics are seldom used in clean surgery for UDT and IH in children in the United States [7]. Thus, consensus on the necessity of preoperative antibiotics remains unclear. Indeed, only a few studies have been published on the administration of preoperative antibiotics in surgery for umbilical hernia (UH) in children.

In addition, owing to problems with side effects and resistant bacteria caused by the administration of antimicrobial agents, the use of these agents is best minimized [8,9]. In the present study, we aimed to clarify the frequency of SSI in patients who underwent surgery for UDT, IH, or UH as well as the necessity of preoperative antibiotics for the prevention of SSI.

## Materials and methods

This study was approved by the ethics committee of Kagawa University Hospital (registration number: 28-166). The need for informed consent was waived owing to the retrospective and observational nature of the study. Computer-based medical records of patients who underwent surgery for UDT, IH, or UH at our hospital between January 2009 and December 2017 were retrospectively examined. The patients who underwent surgery for IH were subdivided based on the surgical form into those who underwent open IH (OIH) repair and those who underwent laparoscopic percutaneous extraperitoneal closure (LPEC). The patients who underwent surgery for UDT or UH, OIH repair, or LPEC were divided into two groups according to whether they received preoperative antibiotics with surgery. We then determined the differences in the SSI incidence within 30 days of surgery. Before 2012, whether preoperative antibiotic treatment was needed was determined at the operator’s discretion. Thereafter, we unified the usage of preoperative antibiotics. In OIH repair and surgeries for UDT, we did not use preoperative antibiotics. Conversely, we principally used a single dose of cefazolin 30 minutes preoperatively in patients who underwent surgery for UH and LPEC. Next, we determined the differences in the SSI incidence rates before and after 2012. We hypothesized that the SSI incidence may be higher in patients who underwent surgery for UH or LPEC, wherein the umbilicus wound was incised than in those who underwent surgery for the inguinal region (surgery for UDT and OIH repair). The relative risk of SSI postoperatively for UH and LPEC, where an umbilical incision was performed, was compared with that after an inguinal incision in surgery for UDT and OIH repair.

When patients simultaneously underwent surgery for UDT or OIH repair bilaterally, we considered the presence of two wounds. For UDT, both traditional inguinal incisions, orchiopexy and single scrotal incision orchiopexy, were performed. Thus, when patients underwent surgery with traditional orchiopexy, we considered the presence of two wounds. We performed conventional LPEC, which necessitates two skin incisions for a camera and grasping forceps. Thus, when patients underwent LPEC, we considered the presence of two wounds. A patient who underwent another surgery on a different day was considered a new patient. We compared the influence of age, gender, height, body weight, operative time, and period of anesthetization. The exclusion criteria were as follows: age of ≥16 years, simultaneous surgery for other diseases, intra-abdominal testis, emergency surgery, and a history of oral administration of antibiotics one week preoperatively.

On the day before surgery, the patients took a shower or bath. They underwent skin disinfection with 10% povidone-iodine at the surgical site when the surgery was initiated. In reference to the CDC guidelines, SSI was defined as follows: the attending physician performed puncture, drainage, and administration of antibiotics after judging the extent of the patient’s wound infection based on physical findings (e.g., pain or tenderness, localized swelling, redness, and fever) or bacterial culture examination within 30 days of surgery [2]. The presence/absence of SSI was evaluated when the patients visited the outpatient department one and four weeks postoperatively.

To determine the significance of the differences, a Fisher’s exact test (SSI incidence, gender, and relative risk) and a Student’s t-test (e.g., age, height, body weight, operative time, and period of anesthetization) were conducted. All statistical analyses were performed with EZR (Saitama, Japan: Saitama Medical Center, Jichi Medical University) [10]. Statistical significance was set at p < 0.05.

## Results

Of the 966 patients diagnosed with UDT, IH, or UH and who underwent surgery at our hospital between January 2009 and December 2017, 926 patients (1389 wounds) were enrolled in this study. Table 1 shows the subjects’ characteristics.

**Table 1:** Baseline characteristics of patients evaluated for SSI. ^a^Fisher’s exact test was done. ^b^Student’s t-test was done. SSI: surgical site infection

Variables	SSI	Non-SSI	p-Value
Total wounds, n (%)	7 (0.5)	1382 (99.5)	n/a
Total patients, n (%)	7 (0.8)	919 (99.2)	n/a
Male, n (%)	6 (1.0)	608 (99.0)	0.43^a^
Female, n (%)	1 (0.3)	311 (99.7)	
Age (years), (mean ± SD)	4.4 ± 2.0	3.5 ± 2.6	0.38^b^
Height (cm), (mean ± SD)	102 ± 16	93 ± 20	0.26^b^
Body weight (kg), (mean ± SD)	18 ± 7.5	15 ± 6.7	0.20^b^
Operative time (min), (mean ± SD)	55 ± 7.8	56 ± 27	0.89^b^
Anesthesia time (min), (mean ± SD)	101 ± 13	102 ± 32	0.92^b^

SSI was observed for a total of seven wounds (0.5%). Regarding the patient factors, no statistically significant differences were noted in the SSI incidence based on gender, age, height, and body weight. Regarding the intervention factors, no statistically significant differences were found based on the operative time and period of anesthetization. SSI rates in patients who underwent surgeries before and after 2012 were 0.7% and 0.4%, respectively; however, the difference was not statistically significant (p = 0.44). Table 2 shows the SSI incidence according to the type of surgical procedure.

**Table 2:** SSI incidence according to the type of surgical procedure. Data are presented as number (%). Fisher’s exact test was done. SSI: surgical site infection; UDT: undescended testis; OIH: open inguinal hernia; LPEC: laparoscopic percutaneous extraperitoneal closure; UH: umbilical hernia

Surgical procedure	Total	Antibiotic given	No antibiotic given	p-Value
Total wounds	7/1389 (0.5)	5/492 (1.0)	2/897 (0.2)	0.10
UDT	1/447 (0.2)	1/187 (0.5)	0/260 (0)	0.42
OIH repair	2/606 (0.3)	0	2/606 (0.3)	1.0
LPEC	1/226 (0.4)	1/220 (0.5)	0/6 (0)	1.0
UH	3/110 (2.7)	3/85 (3.5)	0/25 (0)	1.0

In all, patients with 492 wounds (35%) received preoperative antibiotics. The most common antimicrobial agent used was cefazolin (81%), followed by a combination of cefazolin and third-generation cephalosporins (oral administration; 17%) and cefmetazole (1.8%). A patient who received cefazolin experienced skin rash as a side-effect. Oral antibiotics were administered only to patients with UDT (85 wounds) before 2012, approximately three days postoperatively. No occurrence of SSI was observed in patients in whom oral antibiotics were administered.

In the entire group of patients with UDT, those with 187 wounds (42%) received antibiotics. The total SSI incidence after surgery for UDT was 0.2%, with no statistically significant difference based on the presence/absence of antibiotics. The site of infection was at the scrotal incision. No patient who underwent OIH repair received antibiotics, and the SSI incidence in these patients was 0.3%. Total SSI incidence in patients who underwent LPEC was 0.4%. Several patients who underwent LPEC received antibiotics (97%), and the SSI incidence rate in these patients was 0.5%. The site of infection was at the umbilical incision. In patients who underwent LPEC, the relative risk of SSI compared with that in those who underwent surgery in the inguinal region was not statistically significant at 1.6 (95% CI = 0.030-19; p = 0.54). Among patients who underwent surgery for UH, 85 (77%) patients received antibiotics. The total SSI incidence in surgery for UH was 2.7%. The SSI incidence rate was higher for surgery for UH than for other surgeries. In patients who underwent surgery for UH, the relative risk of SSI compared with that in those who underwent surgery in the inguinal region was statistically significant at 9.8 (95% CI = 1.3-74; p = 0.013). Table 3 shows details of patients with SSI. The median period until the diagnosis of SSI was six days. The site of infection was the incised portion on the surface layer in all patients.

**Table 3:** Characteristics of patients with SSI. SSI: surgical site infection; UDT: undescended testis; OIH: open inguinal hernia; LPEC: laparoscopic percutaneous extraperitoneal closure; UH: umbilical hernia; POD: postoperative day; CEZ: cefazolin; superficial: superficial incisional infection

Patient	Surgical procedure	Age (years)	Sex	POD to infection (days)	Infection type	Antibiotic
1	UDT	7.6	Male	1	Superficial	CEZ
2	OIH repair	3.7	Male	6	Superficial	n/a
3	OIH repair	4.5	Male	10	Superficial	n/a
4	LPEC	5.2	Female	3	Superficial	CEZ
5	UH	1.9	Male	26	Superficial	CEZ
6	UH	2.1	Male	6	Superficial	CEZ
7	UH	5.7	Female	18	Superficial	CEZ

## Discussion

SSI is a relatively common complication, with a reported incidence of 2.4% in overall pediatric surgeries [11], 0.34-2.9% in pediatric urological surgeries with clean wounds (including cases with UDT and IH) [12,13], and 1.4% in surgeries for UH [14]. The total SSI incidence was 0.5% in this study, which is in concordance with that reported by some studies. SSI may result in longer hospital stays, higher hospital charges, and complications. Therefore, appropriate prophylaxis for SSI is essential [1].

One of the measures undertaken for preventing SSI is preoperative antibiotic use. In previous studies, the SSI incidence reportedly decreased with the administration of antibiotics in adult patients undergoing mastectomy, appendectomy, colon surgery, and OIH repair as surgery for IH [1,15]. Conversely, only a few studies on preoperative antibiotic use in children are available. Although guidelines for surgeries for UDT and IH have been published by the American Urological Association and later by the European Association of Urology, both sets of guidelines focus on adults and have excluded pediatric diseases [4,5]. The Japanese Urological Association guidelines have described pediatric surgery. However, the guidelines were not sufficiently examined because the guidelines were based only on the results of a questionnaire study [6].

When considering antibiotic use, issues of side effects and resistant bacteria should also be considered. Side effects caused by perioperative drug administration occur in one in 5,000-25,000 people and are associated with a mortality rate of 6% [8,16]. In addition, antibiotic-related anaphylaxis accounts for 8-28% of side effects caused by perioperative drug administration [8,16]. According to a questionnaire conducted on pediatric urologists in the United States, 61% of respondents considered the possibility of side effects when administering preoperative antibiotics, which indicates their high interest in the side effects [7].

Intestinal Bifidobacterium count is decreased and *Pseudomonas aeruginosa* and Enterococcus counts are increased by the administration of antibiotics for four days, and repeat administration of antibiotics may result in the emergence of resistant bacteria [9,17]. Antibiotics should be used to minimize certain problems like side effects and resistant bacteria.

In a study on pediatric urological surgery by Ellett et al., the SSI incidence was as low as 0.34% in clean wound urinary tract and closed intestine surgeries that were performed in accordance with the CDC guidelines [12]. In addition, clean wound surgery included no significant difference in the SSI incidence depending on whether antibiotics were used, suggesting that antibiotics were unnecessary [12]. Furthermore, in our study, no significant difference was observed in the SSI incidence between patients who underwent surgery for UDT and were receiving or not receiving antibiotic treatment. In patients who underwent OIH repair, those who received no antibiotic showed a low SSI incidence (0.3%); thus, we can conclude that antibiotics were unnecessary in surgery for UDT and OIH repair.

Patients who underwent LPEC and received a single dose of antibiotic showed an acceptable SSI incidence rate (0.5%). Conversely, another study on IH in adults reported a protective efficacy of antibiotics in patients who underwent OIH repair, but not in those who underwent laparoscopic surgery [15]. In the future, further investigations are needed to determine whether a single dose should be administered or if antibiotics are not necessary at all.

The SSI incidence was significantly higher in patients who underwent surgery for UH than in those who underwent surgery in the inguinal region. Thus, UH should be considered as a disease with a high risk of SSI. Indeed, in another study, the SSI incidence in patients who underwent surgery for UH was 1.4%, which was slightly higher than our finding of SSI incidence in patients who underwent surgery for UDT, OIH repair, and LPEC [14]. Moreover, the umbilicus itself was considered to be a high risk because of the moist environment and residential bacteria [18].

However, the umbilicus wound was incised in patients who underwent surgery for both UH or LPEC. SSI incidence differs between surgery for UH and LPEC. We assumed two reasons for this difference. First, the wide area of the subcutaneous tissue exfoliated around the umbilicus, which is required in surgery for UH, may be related to the increased SSI incidence rate. In patients who underwent surgery for hypertrophic pyloric stenosis performed with an upper umbilical incision, a high SSI incidence of 2.3-6.7% has been reported [18,19]. It is necessary to exfoliate a wide range of subcutaneous tissue. Second, surgery for UH may possibly lead to hematoma formation or ischemia because connective tissues of the umbilicus are excised, which relates to the increased SSI.

Because of the high SSI incidence in surgery for UH, antimicrobial agents should be used. However, considering that the SSI incidence was still high despite the administration of antibiotics, the prevention of SSI in patients with UH with antibiotics alone may be difficult. In fact, it has been reported that chlorhexidine-alcohol is superior to povidone-iodine for preventing SSI and that the SSI incidence is decreased with umbilical negative pressure dressing use during transumbilical laparoscopic-assisted appendectomy; thus, additional prophylaxis may be necessary in such cases [20,21].

Limitations

This study has several limitations. First, it was a retrospective study with no control group included. Second, the number of cases was small, particularly for LPEC and surgery for UH, which may have weakened the significance of our findings. Lastly, although there was no statistically significant difference in the SSI incidence rates before and after 2012, the fact that the usage of preoperative antibiotics was not unified was side-lined. To resolve these problems, a prospective randomized controlled trial should be performed with a larger number of subjects in the future.

## Conclusions

There was no significant difference in the SSI incidence between patients who underwent surgery for UDT with antibiotics and those who did not. Patients who underwent OIH repair did not receive antibiotics, but they showed a low SSI incidence (0.3%); thus, we can conclude that preoperative antibiotics were unnecessary in surgery for UDT and OIH repair. Patients who underwent LPEC and received a single dose of antibiotics showed an acceptable SSI incidence (0.5%). In the patients who underwent surgery for UH, the relative risk of SSI compared with that in those who underwent surgery in the inguinal region was statistically significant at 9.8 (95% CI = 1.3-74; p = 0.013). Considering this finding, it is believed that more care should be taken in patients undergoing surgery for UH as they are at higher risk of SSI. Further studies are required in the future to assist our understanding of the prevention of SSI.
